# Forsythiaside attenuates lipopolysaccharide-induced inflammatory responses in the bursa of Fabricius of chickens by downregulating the NF-κB signaling pathway

**DOI:** 10.3892/etm.2013.1378

**Published:** 2013-10-31

**Authors:** GUANGDONG CHENG, YULIAN ZHAO, HE LI, YUE WU, XIANXIAN LI, QIANG HAN, CHONGSHAN DAI, YANHUA LI

**Affiliations:** 1 College of Veterinary Medicine, Northeast Agricultural University, Harbin, Heilongjiang 150030, P.R. China; 2 College of Life Science, Jiamusi University, Jiamusi, Heilongjiang 154007, P.R. China

**Keywords:** forsythiaside, bursa of Fabricius, lipopolysaccharide, inflammation, nuclear factor-κB

## Abstract

Forsythiaside, a phenylethanoside product isolated from air-dried fruits of *Forsythia suspensa*, has been demonstrated to exhibit antioxidant, antibacterial and anti-inflammatory activities *in vitro*. However, its mechanism and the effects of lipopolysaccharide (LPS)-induced injury on the bursa of Fabricius (BF) of chickens are poorly understood. The present study aimed to investigate the anti-inflammatory effects of forsythiaside on LPS-induced acute inflammation. In addition, the potential molecular mechanisms of forsythiaside were analyzed in the BF, a special immune organ in chickens. Forty 15-day-old chickens were randomly divided into control, LPS and LPS plus forsythiaside (30 or 60 mg/kg) groups (n=10 for each group). In the LPS plus forsythiaside (30 or 60 mg/kg) groups, the chickens were orally administered with forsythiaside at doses of 30 and 60 mg/kg for seven days. At 21 days old, the chickens were intravenously injected with 200 μg/kg body weight LPS. Chickens in the control and LPS groups were only administered with vehicle or LPS, respectively, at day 21. At 3 h post-injection, the body temperature and nitric oxide (NO) levels were analyzed. In addition, the levels and mRNA expression of pro-inflammatory cytokines, including tumor necrosis factor-α (TNF-α), interleukin-6 (IL-6) and IL-1β, and the mRNA expression of nuclear factor-κB (NF-κB), cyclooxygenase-2 (COX-2) and inducible NO synthase (iNOS), were examined in the BFs isolated from the chickens. The results revealed that forsythiaside was able to attenuate the LPS-induced inflammatory responses in the BFs of the chickens. The mechanisms by which forsythiaside exerted its anti-inflammatory effect were found to correlate with the inhibition of IL-6, IL-1β, TNF-α and COX-2 production, via the inactivation of NF-κB, indicating that the NF-κB-iNOS-NO signaling pathway may be important in this process.

## Introduction

Lipopolysaccharide (LPS), the major component of the outer membrane of Gram-negative bacteria, also plays a key role in the recognition and signaling responses that lead to the elimination of invading pathogens. The immune system is important for fighting bacterial infections and mediating deleterious host reactions in animals and humans (1–2).

LPS-induced inflammation develops by the secretion of various pro-inflammatory mediators, including tumor necrosis factor-α (TNF-α), interleukin-1β (IL-1β), IL-6, cyclooxygenase-2 (COX-2), inducible nitric oxide synthase (iNOS) and prostaglandin E2 (PGE2) (3). During infections, IL-1β and TNF-α, which are the classic pro-inflammatory cytokines, act first in the inflammation process. Nuclear factor (NF)-κB is downstream of the signaling pathway activating IL-1β and TNF-α. Recent studies have shown that NF-κB is central to the regulation of a number of genes responsible for the generation of inflammatory mediators, for example iNOS and COX-2 (4). The increased activation of NF-κB has been observed in heart, brain, spleen and lung injuries following LPS exposure (5,6).

*Forsythia suspensa* Vahl. (*F. suspensa*) is a well-known Chinese herbal medicine that has been used as an important source of medicine for pyrexia, inflammation, ulcers and gonorrhea (7–9), based on its antioxidant, antibacterial, antiviral, choleretic and antiemetic activity (10–12). Studies have shown that forsythiaside and forsyth from *F. suspensa* constitute the major bioactive components of this plant (13,14). Forsythiaside, a phenylethanoside, has been shown to exhibit antibacterial, antioxidant and antiviral activity *in vivo* and *in vitro*(15). A study by Jiang *et al*(16) showed that forsythiaside reduced serum levels of TNF-α and IL-6, decreased the infiltration of leukocytes and reduced the histopathological damage in a rat myocardial ischemia-reperfusion (I/R) model. In addition, Forsythiaside has been demonstrated to attenuate lipid peroxidation, decrease lipoprotein-induced endothelin-1 secretion by endothelial cells and inhibit COX-2 activity (17–19). However, the effect of forsythiaside on the inflammatory cytokine production induced by LPS in broiler chickens has not been investigated. As well as the liver, spleen and thymus, the bursa of Fabricius (BF) is a primary immune organ and it is also a unique avian humoral immune organ (20). The present study aimed to investigate the anti-inflammatory effect of forsythiaside by examining changes in body temperature and levels of pro-inflammatory cytokines, including IL-1β, IL-6 and TNF-α, induced by LPS in the BFs of broiler chickens. Furthermore, NF-κB, iNOS and COX-2 mRNA expression was examined to further investigate the potential mechanisms involved in the effects of forsythiaside.

## Material and methods

### Chemicals and reagents

Forsythiaside, with a purity of 98.0%, was obtained from Chengdu Herbpurify Co., Ltd. (Chengdu, China) and *Escherichia coli* LPS (L2880; serotype, O55:B5) was obtained from Sigma-Aldrich (St. Louis, MO, USA). ELISA kits for TNF-α, IL-1β and IL-6 were purchased from R&D Systems (Minneapolis, MN, USA), while NO assay kits were obtained from the Nanjing Jiancheng Bioengineering Institute (Nanjing, China). A BCA protein assay kit was purchased from Wuhan Boster Bio-engineering Limited Co. (Wuhan, China) and TRIzol reagent was obtained from Invitrogen Life Technologies, (Carlsbad, CA, USA). Moloney murine leukemia virus (M-MLV), RNase inhibitor, oligo-dT, deoxyribonucleotide triphosphate (dNTP) and 5X buffer were purchased from Takara Biotechnology (Dalian) Co., Ltd. (Dalian, China). A FastStart Universal SYBR Green Master (Rox) was obtained from Roche Diagnostics (Indianapolis, IN, USA).

### Animals and treatment

One-day-old male Arbor Acres broiler birds were obtained from a local hatchery and housed in starter batteries with access to water and commercial feed *ad libitum*, in accordance with NRC recommendations. At 15 days of age, 40 chickens were randomly divided into four treatment groups, control, LPS and LPS plus forsythiaside (30 or 60 mg/kg), with 10 chickens in each group. In the LPS plus forsythiaside (30 or 60 mg/kg) groups, the chickens were orally administered with forsythiaside at doses of 30 and 60 mg/kg body weight (BW), respectively, for seven days. At 21 days of age, the chickens in the LPS and the LPS plus forsythiaside (30 or 60 mg/kg) groups were intravenously injected with LPS at 200 μg/kg BW, while the control group received an equal volume of saline. The study was approved by the Northeast Agricultural University, Harbin, China.

### Determination of cloacal temperature

The cloacal temperature of each bird was measured prior to and 3 h after injection of LPS using a thermocouple rectal probe thermometer. In addition, the general behavioral changes of these birds, including agility and feeding patterns, were also observed following the treatments, prior to sacrifice. The chickens were humanely euthanized by cervical dislocation and the BF was collected from each animal. Each BF was frozen immediately with liquid nitrogen and stored at −80°C until further analysis.

### Sample collection

The isolated BFs were divided into two parts and one part was weighed. Following this, 0.9% saline, measuring nine-fold the weight of the BF tissue, (W:V=1:9) was added to a beaker. The BFs were then minced, ground and centrifuged at 3,000 × g for 10 min. The extracted supernatant, representing a 10% tissue suspension, was stored at −80°C until processing. The remaining part of each BF was isolate RNA.

### Measurement of NO levels

The concentration of NO in the BF tissues was determined using an NO assay kit, according to the manufacturer’s instructions. Briefly, the method involved measuring the levels of NO metabolites, including nitrite and nitrate. Nitrate was reduced first to nitrite by the action of nitrate reductase and the reaction was then initiated by the addition of Griess reagent, prior to the absorbance of the mixture at 550 nm being measured (4).

### Measurement of IL-1β, TNF-α and IL-6 levels

The tissue samples were centrifuged at 3,000 × g(Sigma-Aldrich, St. Louis, MO, USA), for 10 min at 4°C. Following this, the cytokine concentrations of IL-1β, TNF-α and IL-6 in the BFs were assayed using chicken ELISA kits, according to the manufacturer’s instructions.

### Measurement of IL-1β, TNF-α, IL-6, COX-2, NF-κB and iNOS mRNA expression

#### RNA isolation and reverse transcription

Total RNA was isolated using TRIzol reagent, in accordance with the manufacturer’s instructions. Total RNA was subsequently converted to cDNA using 8 μl oligo-dT primers and 8 μl dNTP in 104 μl ddH_2_O at 70°C for 5 min, followed by 32 μl 5X buffer, 4 μl RNase inhibitor and 4 μl M-MLV at 42°C for 1 h. The reaction was terminated by heating at 70°C for 15 min.

#### Quantitative polymerase chain reaction (qPCR)

qPCR was performed using a LightCycler^®^ 480 System (Roche Diagnostics) and the reactions were performed in 96-well plates (Roche Diagnostics) in a volume of 20 μl containing 10 μl LightCycler FastStart DNA Master SYBR Green I, 1.2 μl cDNA, 0.6 μl of each primer and 7.6 μl ddH_2_O. Standard cycling conditions were used, including a pre-amplification step of 95°C for 10 min, followed by amplification for 40 cycles of 95°C for 15 sec, 60°C for 1 min and 72°C for 20 sec. All the samples were analyzed in triplicate. The mean cycle threshold (Ct) was calculated for the target and house-keeping (β-actin) genes. The amount of the target gene was normalized relative to that of the housekeeping gene (ΔCt=Ct_target_ - Ct_housekeeping_). The ΔΔCt value was calculated by subtracting the ΔCt of the non-stimulated sample from the ΔCt of the stimulated sample. The relative amount of the target gene in the stimulated sample to that in the non-stimulated sample was calculated by the 2^−ΔΔCt^ method. The primers used are shown in Table I.

#### Statistical analysis

Quantitative data from the experiments are expressed as the mean ± standard deviation. All groups were compared using a one-way analysis of variance with SPSS 11.5 statistical software (SPSS, Inc., Chicago, IL, USA) and an independent samples t-test. P<0.05 was considered to indicate a statistically significant difference.

## Results

### Clinical changes

**Figure 1:**
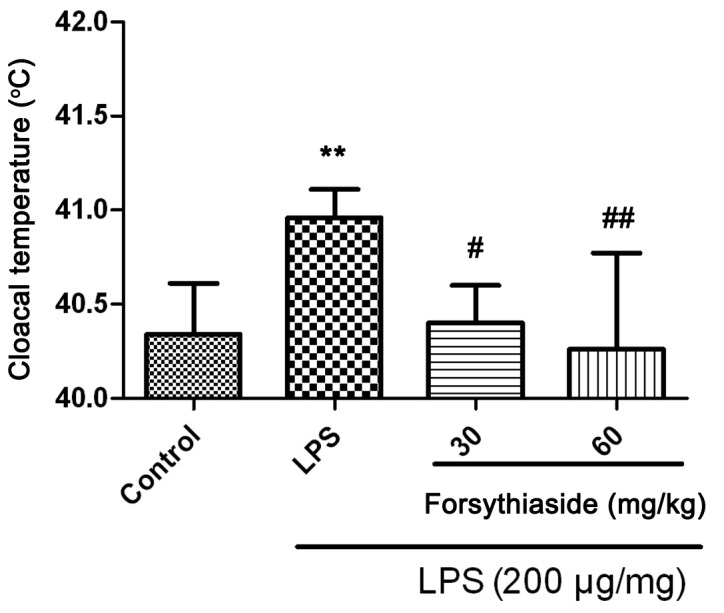
Effect of forsythiaside on the cloacal temperature of LPS-treated chickens. Data are expressed as the mean ± standard deviation (n=10). ^*^P<0.05 and ^**^P<0.01, vs. control group; ^#^P<0.05 and ^##^P<0.01, vs. LPS-treated group. LPS, lipopolysaccharide.

Following LPS treatment, the chickens in the LPS group showed symptoms of drowsiness and lethargy and exhibited ruffled feathers and slight diarrhea within 3 h of injection. These effects were not present in the control group, while in the LPS plus forsythiaside (30 or 60 mg/kg) groups the symptoms were milder than those of the LPS group. In addition, the cloacal temperature of the chickens in the LPS group was elevated at 3 h post treatment, while the 30 or 60 mg/kg forsythiaside pretreatment for seven days appeared to prevent the LPS-induced increase in cloacal temperatures (Fig. 1).

### Change in NO levels in the BFs of the chickens

**Figure 2:**
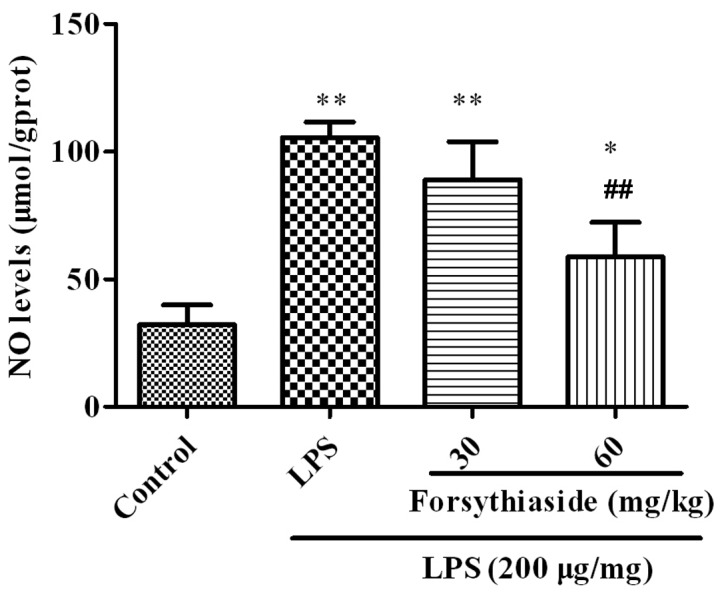
Effect of forsythiaside on the NO levels in the bursa of Fabricius of LPS-treated chickens. Data are expressed as the mean ± standard deviation (n=10). ^*^P<0.05 and ^**^P<0.01, vs. control group; ^#^P<0.05 and ^##^P<0.01, vs. LPS-treated group. NO, nitric oxide; LPS, lipopolysaccharide.

The NO levels were examined in the BFs of the chickens and the results are shown in Fig. 2. In the LPS group, the NO level in the BF was significantly increased to (105.5±6.2 μmol/g protein), compared with the control group (P<0.01). When the chickens were administered forsythiaside for seven days prior LPS injection, i.e., in the LPS plus forsythiaside (30 or 60 mg/kg) groups, the NO levels were significantly decreased to 89.2±14.9 and 58.7±136 μmol/g protein, respectively, compared with the LPS alone group (P<0.01).

### Changes in IL-1β, IL-6 and TNF-α levels in the BFs of the chickens

**Figure 3:**
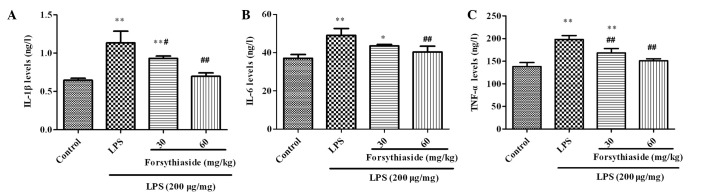
Effect of forsythiaside on the IL-1β, IL-6 and TNF-α levels in LPS-treated chickens: (A) IL-1β, (B) IL-6 and (C) TNF-α. Data are expressed as the mean ± standard deviation (n=10). ^*^P<0.05 and ^**^P<0.01, vs. control group; ^#^P<0.05 and ^##^P<0.01, vs. LPS-treated group. IL, interleukin; TNF, tumor necrosis factor; LPS, lipopolysaccharide.

The concentrations of IL-1β, IL-6 and TNF-α in the BF were examined using ELISA and the results are shown in Fig. 3. Three hours after LPS injection, the levels of the cytokines, IL-1β, IL-6 and TNF-α, in the BF homogenate were markedly increased compared with those in the control group. As shown in Fig. 3, pretreatment with forsythiaside (30 or 60 mg/kg) significantly decreased the levels of IL-1β, IL-6 and TNF-α in a dose-dependent manner.

### Changes in IL-1β, TNF-α, IL-6, COX-2, NF-κB and iNOS mRNA expression

**Figure 4:**
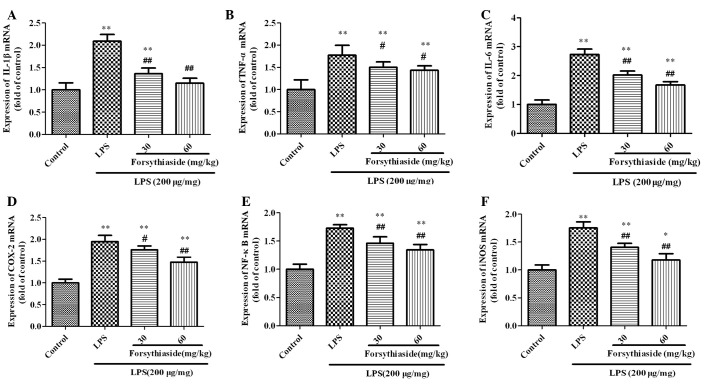
Effect of forsythiaside on the IL-1β, TNF-α, IL-6, COX-2, NF-κB and iNOS mRNA expression in the bursa of Fabricius of LPS-treated chickens: (A) IL-1β, (B) TNF-α, (C) IL-6, (D) COX-2, (E) NF-κB and (F) iNOS. Data are expressed as the mean ± standard deviation (n=10). ^*^P<0.05 and ^**^P<0.01, vs. control group; ^#^P<0.05 and ^##^P<0.01,vs. LPS-treated group. IL, interleukin; TNF, tumor necrosis factor; LPS, lipopolysaccharide; COX-2, cyclooxygenase-2; NF-κB, nuclear factor-κ; iNOS, inducible nitric oxide; LPS, lipopolysaccharide.

The mRNA expression of IL-1β, TNF-α, IL-6, COX-2, NF-κB and iNOS in the BF was examined and the results are shown in Fig. 4. Three hours after LPS injection, the mRNA expression of IL-1β, TNF-α, IL-6, COX-2, NF-κB and iNOS in the BF homogenate of the LPS group were significantly increased to 2.1±0.15-, 1.77±0.23-, 2.73±0.19-, 1.95±0.14-, 1.73±0.07- and 1.75±0.14-fold the expression levels of the control group, respectively. However, pretreatment with forsythiaside (30 or 60 mg/kg) significantly decreased the levels of IL-1β, TNF-α, IL-6, COX-2, NF-κB and iNOS mRNA expression compared with the LPS alone group in a dose-dependent manner.

## Discussion

In the present study, the effects of forsythiaside on the acute-phase response to LPS-induced inflammation in the BFs of broiler chickens were measured. Our results demonstrated that forsythiaside exhibits a promising anti-inflammatory activity by decreasing cloacal temperature and the manifestation of clinical symptoms. In addition, these protective effects were found to correlate with the attenuation of the inflammatory responses. The *in vitro* anti-inflammatory effects of forsythiaside have been reported in a previous study (13). However, to the best of our knowledge, this study has demonstrated for the first time that forsythiaside is able to protect against LPS-induced injury in the BF of the chicken.

In the present study, when the chickens were administered with LPS alone, the cloacal temperature of the chickens was significantly increased, compared with the control group, and specific abnormal symptoms were apparent. These observations are consistent with previous studies (21–23). However, in the chickens that were pretreated with forsythiaside, these symptoms and increases in cloacal temperature were reduced. IL-1β, IL-6 and TNF-α are the primary mediators of the acute-phase response (21,24,25). It is known that LPS stimulation leads to the production of the pro-inflammatory cytokines, IL-1β, IL-6 and TNF-α, in chicken organs, including the spleen, liver and BF (3,20,22,23). Increases in the levels of these cytokines were observed 3 h after intravenous injection of 200 μg/kg BW LPS and were reversed in chickens pretreated with forsythiaside (30 or 60 mg/kg).

NO is a highly reactive free radical involved in a number of physiological and pathological processes in the inflammatory reaction (26). It is produced by iNOS and reacts with superoxide to yield peroxynitrite, particularly in immune cells. iNOS expression is associated with the upregulation of NF-κB, and NF-κB sites identified in the iNOS gene promoter region, which can be activated by LPS (1). In the present study, the levels of NF-κB mRNA were significantly elevated at 3 h after injection of LPS compared with the control group. The reduction in NO production in the BFs treated with forsythiaside is likely to be relevant to these observations and may be linked to alterations in the signaling cascades triggered by iNOS expression. These results have demonstrated that the anti-inflammatory effects of forsythiaside may be mediated by the NF-κB-iNOS-NO signaling pathway. In addition, the iNOS-NO signaling pathway may also have contributed to the oxidative stress induced by LPS, which, in the LPS plus forsythiaside group, was downregulated due to the antioxidative effects of forsythiaside (13,27).

The NF-κB signaling pathway is regulated by a number of different factors or signaling pathways, including IL-1β, TNF-α, caspase-3, reactive oxygen species p38, c-Jun N-terminal kinases and extracellular signal-regulated kinases/mitogen-activated protein kinases (28,29). Inflammation and oxidative stress are mutual influences in specific diseases and NF-κB may be pivotal to this relationship (30). The activation of NF-κB increases the expression of specific inflammatory factors, including COX-2, IL-8 and TNF-α (29). In the present study, there was a marked inhibition of IL-6, IL-1β, TNF-α and COX-2 secretion in the BFs of chickens that were pretreated with forsythiaside, which may be attributable to the effects of forsythiaside on NF-κB action (31). Jiang *et al*(16) revealed that forsythiaside B decreased inflammatory mediators, including NF-κB, TNF-α and IL-6, in a rat myocardial I/R injury model.

In conclusion, results of the current study indicate that forsythiaside reduces LPS-induced injury in the BFs of chickens, due to its anti-inflammatory function. The mechanisms by which forsythiaside exerts its anti-inflammatory effect correlate with the inhibition of IL-6, IL-1β, TNF-α and COX-2 production, via the inactivation of NF-κB. In addition, the NF-κB-iNOS-NO signaling pathway may be important in this process. This study provide further insight into the anti-inflammatory mechanisms of forsythiaside.

## Figures and Tables

**Table I:** Primer sequences for the real-time polymerase chain reaction used in this study.

Gene name	Gene bank accession number	Primer sequence (5′-3′)	Production length, bp
TNF-α	GU230788.1	Forward: GCC CTT CCT GTA ACC AGAT G	71
		Reverse: ACA CGA CAG CCA AGT CAA CG	
iNOS	NM_204961	Forward: CCT GGA GGT CCT GGA AGA GT	82
		Reverse: CCT GGG TTT CAG AAG TGG C	
NF-κB p50	M86930	Forward: TCA ACG CAG GAC CTA AAG ACA T	162
		Reverse: GCA GAT AGC CAA GTT CAG GAT G	
COX-2	NM_001167718.1	Forward: TGT CCT TTC ACT GCT TTC CAT	84
		Reverse: TTC CAT TGC TGT GTT TGA GGT	
IL-6	NM-204628	Forward: AAA TCC CTC CTC GCC AAT CT	106
		Reverse: CCC TCA CGG TCT TCT CCA TAA A	
IL-1β	Y15006.1	Forward: ACT GGG CAT CAA GGG CTA CA	142
		Reverse: GCT GTC CAG GCG GTA GAA GA	
β-actin	L08165	Forward: CAC CAC AGC CGA GAG AGA AAT	135
		Reverse: TGA CCA TCA GGG AGT TCA TAG C	

TNF-α, tumor necrosis factor-α; iNOS, inducible nitric oxide synthase; NF-κB p50, nucelar factor-κB p50; COX-2, cyclooxygenase-2; IL, interleukin.
